# Usefulness of an S-1 dosage formula: an exploratory analysis of randomized clinical trial (JCOG1001)

**DOI:** 10.1007/s10120-022-01315-8

**Published:** 2022-06-29

**Authors:** Takeshi Kawakami, Junki Mizusawa, Hiroko Hasegawa, Hiroshi Imazeki, Kazuki Kano, Yuya Sato, Satoru Iwasa, Shuji Takiguchi, Yukinori Kurokawa, Yuichiro Doki, Narikazu Boku, Takaki Yoshikawa, Masanori Terashima

**Affiliations:** 1 Division of Gastrointestinal Oncology, Shizuoka Cancer Center, Shizuoka, Japan; 2 Japan Clinical Oncology Group Data Center, National Cancer Center Hospital, Tokyo, Japan; 3 Department of Gastroenterology and Hepatology, National Hospital Organization, Osaka National Hospital, Osaka, Japan; 4 Clinical Trial Promotion Department, Chiba Cancer Center, Chiba, Japan; 5 Department of Gastrointestinal Surgery, Kanagawa Cancer Center, Yokohama, Japan; 6 Department of Gastrointestinal Surgery, Tokyo Medical and Dental University, Tokyo, Japan; 7 Department of Gastrointestinal Medical Oncology, National Cancer Center Hospital, Tokyo, Japan; 8 Department of Gastroenterological Surgery, Nagoya City University Graduate School of Medical Science, Nagoya, Japan; 9 Department of Gastroenterological Surgery, Osaka University Graduate School of Medicine, Osaka, Japan; 10 Department of Oncology and General Medicine, The Institute of Medical Science Hospital, The University of Tokyo, Tokyo, Japan; 11 Department of Gastric Surgery, National Cancer Center Hospital, Tokyo, Japan; 12 Division of Gastric Surgery, Shizuoka Cancer Center, Shizuoka, Japan

**Keywords:** Adjuvant chemotherapy, Dosage formula, Gastric cancer, Renal function, S-1

## Abstract

**Background:**

The blood concentration of S-1 and adverse events are affected by renal function. Herein, an S-1 dosage formula was developed based on renal function, indicating the dose for a target blood concentration. This study aimed to explore the usefulness of the formula in adjuvant chemotherapy for gastric cancer.

**Methods:**

In this ad hoc analysis of the JCOG1001 trial, which evaluated the role of bursectomy for resectable gastric cancer, the recommended dose of S-1 was calculated using the following formula: 1447.8 × (14.5 + 0.301 × CLcr + 8.23 × SEX [male = 1, female = 0]) × body surface area (BSA) (mg/day). Patients were divided into three groups by comparing the initial S-1 dose determined using BSA with the dose recommended by the formula: underdose (UD), equal dose (ED), and overdose (OD).

**Results:**

Among 686 eligible patients, 58, 304, and 324 patients were classified into the UD, ED, and OD groups. The patients’ characteristics in the UD/ED/OD groups were median age (53.5/64.0/67.5 years), male sex (98.3%/75.3%/58.0%), and median BMI (24.8/22.8/22.3), respectively. The planned 1-year adjuvant S-1 therapy was completed in 74.1%/73.7%/68.5%, dose reduction was required in 8.6%/21.1%/30.6%, and treatment schedule was altered in 8.6%/17.1/19.8% in the UD/ED/OD groups, resulting in the 5-year overall survival rates of 77.3%/74.3%/77.0%, respectively. The incidences of grade > 3 anemia, thrombocytopenia, diarrhea, stomatitis, and anorexia were significantly higher in the OD group than in the ED and UD groups.

**Conclusions:**

Dose optimization using an S-1 dosage formula can potentially reduce grade ≥ 3 adverse events for overdosed patients.

**Supplementary Information:**

The online version contains supplementary material available at 10.1007/s10120-022-01315-8.

## Introduction

Gastric cancer is the fourth leading cause of cancer-related deaths worldwide [1] and is the third leading cause of cancer-related deaths in Japan [2]. For locally advanced resectable gastric cancer, perioperative chemotherapy and curative surgery are recognized as standard treatments because of the high recurrence rate in surgery alone. In Asia, postoperative adjuvant chemotherapy after a curative resection is the standard of care. The ACTS–GC trial showed a survival benefit of adjuvant S-1 monotherapy for 1 year after the curative resection of pathological stage II/III gastric cancer [3]. The JACCRO-GC-07 trial showed a significant prolongation of overall survival (OS) using adjuvant chemotherapy with S-1 plus docetaxel compared with S-1 monotherapy for patients with pathological stage III gastric cancer [4]. Several studies demonstrated that the completion of a planned adjuvant S-1 therapy is associated with favorable survival outcomes of patients with gastric cancer after a curative resection [5–7]. However, when severe adverse events occur, the treatment is interrupted and terminated. To achieve completion, an appropriate dose reduction and/or treatment schedule alteration are required in some patients.

S-1 is a pro-drug of 5-fluorouracil (5-FU), consisting of tegafur, gimeracil (CDHP), which is an inhibitor of dihydropyrimidine dehydrogenase catabolizing 5-FU, and oteracil. Since more than 50% of CDHP is excreted by the kidneys, its excretion is reduced in patients with low renal function, resulting in persistently high blood 5-FU concentration [8–10] and high frequency of adverse events. Therefore, it is recommended to reduce its dose in cases with low renal function [11]. A low renal function was considered as one of the risk factors for poor compliance of adjuvant chemotherapy with S-1 [12]. In contrast, for cases with good renal function and large body surface area (BSA), the highest approved S-1 dose (120 mg/day) may not be sufficient to achieve the target AUC, and its efficacy may be reduced. It is also important to determine the appropriate S-1 dose for such patients. However, there is no established method, such as the Calvert formula for carboplatin, for patients’ renal function. Therefore, to obtain the target pharmacokinetics of S-1, the S-1 dose is determined according to BSA and is reduced based on the clinical experiences of patients in the clinical practice.

Booka et al. developed a formula for determining the S-1 dosage based on renal function [13]. The validation of this formula led to the development of a revised formula for the determination of the S-1 dosage, which considered the gender of the patient. The revised formula is believed to be useful in metastatic settings [14]; however, it has not been validated in adjuvant settings.

The Japan Clinical Oncology Group (JCOG) study JCOG1001 is a randomized phase 3 trial that investigated the role of bursectomy for cT3/T4 resectable gastric cancer [15], which did not show survival benefits. The enrolled patients received adjuvant S-1 therapy for 1 year as a protocol treatment. The data regarding the clinical courses during adjuvant chemotherapy was collected. This study aimed to explore the clinical utility of the S-1 dosage formula in adjuvant chemotherapy with S-1 for gastric cancer using the data of JCOG1001.

## Patients and method

### Patients

The details of the JCOG1001 trial, registered in UMIN–CTR (UMIN000003688), were previously reported [15]. The main inclusion criteria were histologically proven resectable gastric adenocarcinoma with an estimated cT3 (SS) or cT4 (SE). Patients were randomized into the non-bursectomy arm or bursectomy arm. After R0 resection, the patients received postoperative adjuvant chemotherapy with S-1 as a protocol treatment except for pT1. The severity of adverse events was evaluated by the National Cancer Institute–Common Terminology Criteria for Adverse Events (NCI–CTCAE) version 4.0.

Between June 2010 and March 2015, 1,204 patients were enrolled in the JCOG1001 trial. On the second planned interim analysis on September 17, 2016, when all the protocol treatment, including adjuvant chemotherapy, was finished in all patients, the JCOG Data and Safety Monitoring Committee independently reviewed the results and recommended early terminations for futility, because the OS was lower in the bursectomy group than in the non-bursectomy group, associated with a 12.7% predictive probability of showing superiority. After the publication of the primary results [15], the survival was followed. The cutoff date of this post-hoc study was on April 17, 2020. All clinical data for this ad hoc analysis were obtained from the JCOG1001 database.

The study protocol of the JCOG1001 trial, including the secondary use of trial data, was approved by the JCOG Protocol Review Committee and the institutional review boards of all participating institutions.

### Adjuvant chemotherapy with S-1

An initial S-1 dose was determined on the basis of BSA, which was calculated using the body weight measured at the initiation of the adjuvant S-1 therapy: 80 mg/day for BSA < 1.25 m^2^, 100 mg/day for 1.25 m^2^ ≤ BSA < 1.50 m^2^, and 120 mg/day for BSA ≥ 1.50 m^2^. S-1 was orally administered twice a day for 4 weeks, followed by a 2-week rest period in a 6-week cycle; this was repeated for 1 year after the surgery except in cases, where patients were refractory or intolerant to S-1 therapy or in cases of patients’ refusal. The treatment was interrupted if any of the following adverse events occurred: neutrophil counts < 1000 mm^3^/µL; white blood cell counts < 2000 mm^3^/µL; platelet counts < 50,000 mm^3^/µL; aspartate aminotransferase or alanine aminotransferase > 100 IU/L; total bilirubin > 2.0 mg/dL; creatinine > 1.5 mg/dL; grade 2 or 3 diarrhea, nausea, vomiting, anorexia, mucositis oral, or fever; or any other grade 3 non-hematological toxicities. If the patients experienced adverse events requiring treatment interruption, the dose was reduced, and/or the treatment schedule was altered to a 2-week administration, followed by a 1-week rest repeated twice for one course. The dose of S-1 was not modified only by body weight change without any adverse events. Treatment completion was defined as the continuation of S-1 for 1 year after surgery. The relative dose intensity was calculated as a proportion of the actual dose divided by the planned dose (protocol dose or recommended dose).

### Classification by comparing the doses actually administered and recommended by the S-1 formula

For each patient, the S-1 dose was calculated using the following formula: 1447.8 × (14.5 + 0.301 × CLcr + 8.23 × SEX [male = 1, female = 0]) × BSA (mg/day) [14]. Then, the dosage obtained from the formula was converted into a tablet dosage by applying it to nomogram; this tablet dosage was defined as the recommended dose. The patients were divided into three groups by comparing the initial actually administered dose per protocol with the recommended dose: overdose (OD), equal dose (ED), and underdose (UD) groups. For example, in cases of a recommended dose of 100 mg, if the patients actually received 80 mg, 100 mg, or 120 mg, they were classified as UD, ED, or OD, respectively (Supplementary Table S1).

### Statistical analysis

Categorical parameters were compared using the Chi-square test, whereas continuous variables were compared using the Wilcoxon rank-sum test. Trend test was used for adverse events. Relapse-free survival (RFS) was defined as the duration between the date of randomization and the first disease recurrence or death from any causes. OS was defined as the duration between the date of randomization and the death from any cause. OS and RFS were estimated using Kaplan–Meier method. Multivariable Cox regression analysis was performed to compare the survival rate of the three groups, with adjustments for age, sex, BMI, ECOG PS, tumor diameter, histology, surgical procedure, extent of lymphadenectomy, pT, and pN. All reported *P* values < 0.05 were considered statistically significant. The analyses were performed using SAS version 9.4.

## Results

### Patients’ characteristics

**Fig. 1:**
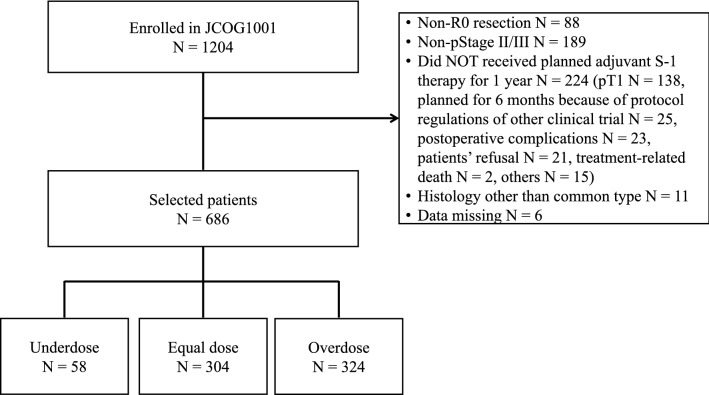
Flow chart of patients

Among 1204 patients enrolled in JCOG1001, 686 patients were selected and classified into the UD group (*n* = 58), ED group (*n* = 304), and OD groups (*n* = 324) (Fig. 1). The comparison between the recommended and actual doses is shown in supplementary Table S1. The patients’ characteristics are shown in Table 1. The median age in the UD, ED, and OD groups was 53.5 (29–77) years, 64.0 (29–80) years, and 67.5 (40–80) years, respectively. The proportions of male patients were 98.3% (*n* = 57), 75.3% (*n* = 229), and 58.0% (*n* = 188), while the median BMI was 24.8 (18.7–29.4), 22.8 (15.0–29.9), and 22.3 (15.5–29.5) in the UD, ED, and OD groups, respectively. There were significantly younger, more male, and more patients with higher BMI in the UD group than in the ED and OD groups.

**Table 1:** Patients’ characteristics

Variables	UD *N* = 58	ED *N* = 304	OD *N* = 324	*P* value
Age, median	53.5	64	67.5	< 0.0001
(range)	(29–77)	(29–80)	(40–80)	
Sex, *n* (%)				
Male	57 (98.3)	229 (75.3)	188 (58.0)	< 0.0001
Female	1 (1.7)	75 (24.7)	136 (42.0)	
ECOG PS, *n* (%)				
0	58	290 (95.4)	309 (95.4)	0.247
1	0	14 (4.6)	15 (4.6)	
BMI, median	24.8	22.8	22.3	< 0.0001
(range)	(18.7–29.4)	(15.0–29.9)	(15.5–29.5)	
Histology, *n* (%)				
Undifferentiated	38 (65.5)	172 (56.6)	168 (51.9)	0.123
Differentiated	20 (34.5)	132 (43.4)	156 (48.1)	
Tumor diameter, median [cm]	5.6	5.5	5.5	0.939
(range)	(2.9–11.5)	(1.5–17.0)	(1.8–14.0)	
Surgical procedure, *n* (%)				
Distal gastrectomy	39 (67.2)	192 (63.2)	216 (66.7)	0.615
Total gastrectomy	19 (32.8)	112 (36.8)	108 (33.3)	
pT, *n* (%)				
T1–T2	7 (12.1)	34 (11.2)	37 (11.4)	0.989
T3	27 (46.5)	136 (44.7)	141 (43.5)	
T4	24 (41.4)	134 (44.1)	146 (45.1)	
pN, *n* (%)				
N0	6 (10.3)	29 (9.5)	43 (13.3)	0.504
N1	12 (20.7)	83 (27.3)	83 (25.6)	
N2	40 (69.0)	192 (63.2)	198 (61.1)	
pStage, *n* (%)				
II	17 (29.3)	104 (34.2)	112 (34.6)	0.733
III	41 (70.7)	200 (65.8)	212 (65.4)	

### Treatment exposure

The proportion of patients who required treatment interruption, schedule alteration, or termination due to adverse events was the smallest in the UD group, followed by that in the ED group and OD group (Table 2). The median relative dose intensity compared with the planned dose in the protocol treatment was 100% in the UD group, 93.8% in the ED group, and 80.7% in the OD group. The median relative dose intensity compared with the dose recommended by the formula was 75.0%, 93.8%, and 108.8%, respectively. The proportion of completion of the planned S-1 therapy for 1 year was similar for the ED (73.7%) and UD (74.1%) groups; however, the OD (68.5%) group had the lowest.

**Table 2:** Treatment exposure

	UD *N* = 58	ED *N* = 304	OD *N* = 324
Treatment interruption, *n* (%)			
Skip	4 (6.9)	79 (25.9)	107 (33.0)
Delay	14 (24.1)	116 (38.1)	103 (31.7)
Dose reduction	5 (8.6)	64 (21.1)	99 (30.6)
Treatment schedule alteration, *n* (%)	5 (8.6)	52 (17.1)	64 (19.8)
Treatment termination, *n* (%)			
Refractory	5 (8.6)	20 (6.6)	18 (5.6)
Intolerance	7 (12.1)	56 (18.4)	81 (25.0)
Patients’ refusal	0	0	1 (0.3)
Others	3 (5.2)	4 (1.3)	2 (0.6)
Median relative dose intensity by the protocol (%)	100	93.8	80.7
(range)	(10.7–133.3)	(7.1–102.5)	(1.8–111.4)
Median relative dose intensity by the formula (%)	75.0	93.8	108.8
(range)	(8.0–111.1)	(7.1–102.5)	(17.9–150.0)
Completion of planned S-1 therapy (%)	74.1	73.7	68.5

### Adverse events

Adverse events ≥ grade 3 in each group are shown in Table 3. The frequency of adverse events ≥ grade 3 was the highest in the OD group followed by the ED and UD groups. As for hematological adverse events, anemia and thrombocytopenia occurred significantly more frequently in the OD group than in the ED and UD groups. No grade ≥ 3 non-hematological toxicities were observed in the UD group, and the OD group had a higher frequency of grade ≥ 3 non-hematological toxicities than the ED group.

**Table 3:** Incidence of ≥ Grade3 adverse events

	UD *N* = 58 (%)	ED *N* = 304 (%)	OD *N* = 324 (%)	Two-sided *P* by trend test
Hematological				
Neutropenia	3 (5.2%)	46 (15.2%)*	53 (16.4%)	0.080
Anemia	0	5 (1.6%)	13 (4.0%)	0.024
Thrombocytopenia	0	0	6 (1.9%)	0.018
Non-hematological				
Fatigue	0	4 (1.3%)	9 (2.8%)	0.082
Diarrhea	0	7 (2.3%)	18 (5.6%)	0.008
Nausea	0	1 (0.3%)	2 (0.6%)	0.448
Stomatitis	0	1 (0.3%)	7 (2.2%)	0.030
Anorexia	0	7 (2.3%)	28 (8.6%)	< 0.0001

*One patient is missing

### OS and RFS

**Fig. 2:**
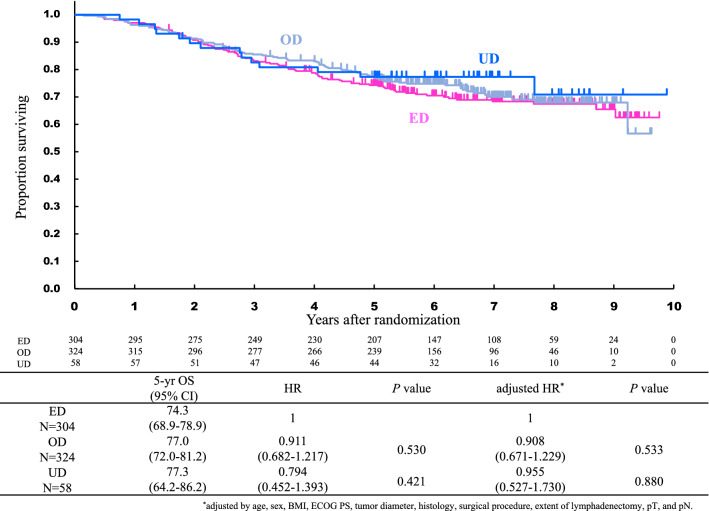
Overall survival of patients with UD, ED, and OD. *UD* underdose, *ED* equal dose, *OD* overdose, *HR* hazard ratio, *CI *confidence interval

**Fig. 3:**
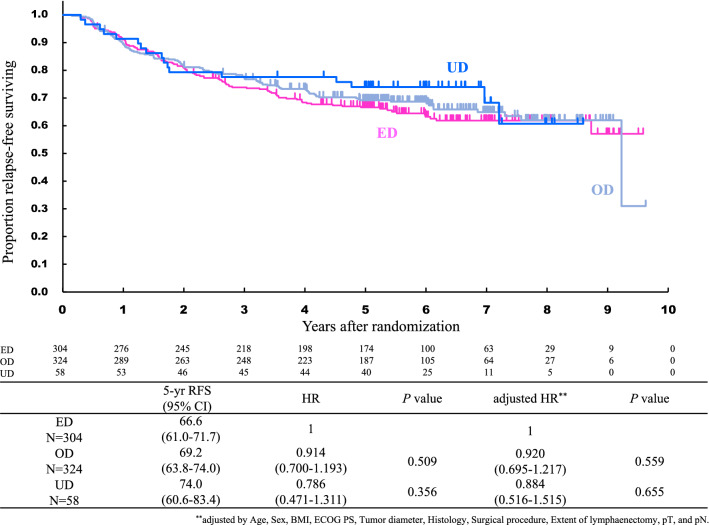
Relapse-free survival of patients with UD, ED, and OD. *UD* underdose, *ED* equal dose, *OD* overdose, *HR* hazard ratio, *CI *confidence interval

There was no significant difference in the 5-year OS rates among the three groups (77.3%, 74.3%, and 77.0% in the UD, ED, and OD groups, respectively). The hazard ratio (HR) for the OD group versus ED group was 0.911 [95% confidence interval (CI), 0.682–1.217], and that for the UD group versus ED group was 0.794 (95% CI, 0.452–1.393) (Fig. 2). The adjusted HR for the OD group versus ED group was 0.908 (95% CI, 0.671–1.229), and that for the UD group versus ED group was 0.955 (95% CI, 0.527–1.730). There was also no significant difference in the 5-year RFS rates among the three groups (74.0%, 66.6%, and 69.2% in the UD, ED, and OD groups, respectively). The HR for the OD group versus ED group was 0.914 (95% CI, 0.700–1.193), and that for the UD group versus ED group was 0.786 (95% CI, 0.471–1.311) (Fig. 3). The adjusted HR for the OD group versus ED group was 0.920 (95% CI, 0.695–1.217), and that for the UD group versus ED group was 0.884 (95% CI, 0.516–1.515).

## Discussion

In summary, this study demonstrates that the OD group had the highest frequency of grade ≥ 3 adverse events and the lowest proportion of completion of adjuvant chemotherapy with S-1 for 1 year, but that there was no difference in RFS and OS among the three groups.

Although it was expected that the proportion of completion of adjuvant S-1 would be the highest in the UD group and the lowest in the OD group, the proportion of completion in the UD group (74.1%) and ED group (73.7%) was similar, and it was the lowest in the OD group (68.5%). Dose reduction and/or treatment schedule alteration are usually performed after the occurrence of grade ≥ 3 adverse events. Because appropriate management also had a positive impact on completion, the impact of dose optimization by the S-1 formula dosage might be reduced in this study. However, considering that severe adverse events were most frequently observed in the OD group at the early phase of adjuvant therapy, which required dose reduction or treatment schedule alteration, and that there was no significant difference in the OS and RFS of the three groups, dose optimization using this formula may be useful to reduce the toxicities in patients classified as the OD group without interfering with its efficacy in clinical practice.

In the palliative setting of S-1 monotherapy (SPIRITS trial [16]), Booka et al. compared the survival outcomes of patients with stage IV gastric cancer classified into the three dose groups by the S-1 dosage formula. It was reported that the OS in the ED group was longer than that in the OD and UD groups [17]. In this study, reproducibility was expected in adjuvant settings. However, there were no significant differences among the three groups, and the survival of the UD group was longer than that of the other groups. There are some reasons to be considered for this discrepancy. First, it is probably due to patient background differences. More patients, who were younger, male, and had higher BMI, were considered to have a better long-term prognosis independently from the efficacy of adjuvant chemotherapy [3, 18]; they were classified into the UD group due to the nature of the formula. Second, patients often experience body weight loss or lowering renal function during adjuvant treatment. As a result, patients who were classified into the UD group might be switched to the ED group because of the body weight loss during treatment. Therefore, it is considered that dose optimization by this formula may be necessary to be repeated according to the patient’s condition. In contrast, while grade ≥ 3 adverse events were observed most frequently in the OD group, the dose of S-1 in the OD group was reduced once adverse events occurred. It was speculated that dose reduction could adjust the S-1 dose in the early period after initiating the adjuvant chemotherapy in the OD group, resulting in treatment completion. Moreover, schedule alteration could reduce the toxicities and maintain the dose intensity regardless of the S-1 dose. Schedule alteration might also reduce the impact of S-1 dose optimization using the S-1 formula on the survival outcomes. The S-1 dosage formula was originally developed for patients with stage IV gastric cancer, and it is unclear whether the formula can be sufficiently extrapolated to adjuvant settings. In this study, even patients in the ED group were required to reduce dosage or skip treatment more frequently compared with patients with advanced gastric cancer who were treated with S-1 monotherapy in the ED group of the SPIRITS trial. The pharmacokinetics of S-1 showed no clinically significant difference immediately before and after gastrectomy [18]. Moreover, during the development of the S-1 formula, gastrectomy showed no relevant impact on the pharmacokinetics of S-1 in patients with unresectable and recurrent gastric cancer [13]. Therefore, it is possible to pharmacokinetically extrapolate this formula to the gastrectomized patients. However, as opposed to the results of the SPIRITS trial, the patients in this study often experienced body weight loss after gastrectomy as described above. Hence, the maintenance of body weight through nutrition support and application of this formula, which is based on the on-time body weight following gastrectomy, might be helpful in reducing toxicities caused by S-1 in adjuvant settings.

At present, the treatment guidelines for gastric cancer recommend S-1 plus docetaxel or oxaliplatin combination therapy, with S-1 or capecitabine as postoperative adjuvant therapy for patients with stage III gastric cancer. The frequency of adverse events tends to be higher in doublet therapy containing S-1 than in S-1 monotherapy. In the aforementioned study on the usefulness of the S-1 formula in patients with stage IV gastric cancer in the G-SOX trial [17], the frequency of adverse events tended to be higher in the OD group than in the ED group. In particular, neutropenia, anemia, and creatinine levels were significantly higher in the OD group than in the ED group. As the dose optimization of S-1 using the S-1 formula may potentially reduce the adverse events of doublet chemotherapy in patients with stage IV gastric cancer, it is expected that dose optimization using this formula is useful for reducing the toxicities of doublet chemotherapy in adjuvant settings.

This study has several limitations. First, body weight often changes during adjuvant treatment; therefore, the formula might not work accurately. Second, the patients’ backgrounds were not balanced among the three groups. As described above, patients who were younger, males, and had higher BMIs tended to be classified in the UD group, whereas those who were older, females, and had lower BMIs tended to be classified in the OD and UD groups due to the nature of the formula. It is believed that these differences in the patients’ background characteristics have some impact on the clinical outcomes. Although it is difficult to adequately avoid this bias, the hazard ratios adjusted for patients’ background characteristics were also calculated in the multivariate analysis for survival. Considering the similar survival among the three groups in the multivariate analysis, it is suggested that the dose optimization by the S-1 formula, especially for the OD group, might reduce the toxicities without interfering with OS and RFS. Finally, because this formula was only developed for Japanese patients, the results of this study cannot be extrapolated to patients in Europe and the United States who show different pharmacodynamics of S-1 from Asian patients.

In conclusion, dose optimization using the S-1 dosage formula may potentially reduce grade ≥ 3 adverse events for the elderly, lower BMI, or female patients who may be overdosed. For adjuvant settings, it may be recommended to use this S-1 dosage formula, considering the chronological changes in body weight and renal function.

## Supplementary Information

Below is the link to the electronic supplementary material.

Supplementary file1 (DOCX 23 KB)
