# Supplementary material for: Usefulness of an S-1 dosage formula: an exploratory analysis of randomized clinical trial (JCOG1001)
Source: Gastric Cancer. 2022 Jun 29;25(6):1073–81. doi: 10.1007/s10120-022-01315-8 (PMC9587934; doi:10.1007/s10120-022-01315-8)
Supplement: Supplementary file 1 — Supplementary file1 (DOCX 23 KB) [file 10120_2022_1315_MOESM1_ESM.docx]

**Title:** Usefulness of an S-1 dosage formula: An exploratory analysis of randomized clinical trial (JCOG1001)

**Journal name:** Gastric Cancer

**Authors:** Takeshi Kawakami^1^, Junki Mizusawa^2^, Hiroko Hasegawa^3^, Hiroshi Imazeki^4^, Kazuki Kano^5^, Yuya Sato^6^, Satoru Iwasa^7^, Shuji Takiguchi^8^, Yukinori Kurokawa^9^, Yuichiro Doki^9^, Narikazu Boku^10^, Takaki Yoshikawa^11^, Masanori Terashima^12^

**Affiliations:** 1. Division of Gastrointestinal Oncology, Shizuoka Cancer Center, 2. Japan Clinical Oncology Group Data Center, National Cancer Center Hospital, 3. Department of Gastroenterology and Hepatology, National Hospital Organization, Osaka National Hospital, 4. Clinical Trial Promotion Department, Chiba Cancer Center, 5. Department of Gastrointestinal Surgery, Kanagawa Cancer Center, 6. Department of Gastrointestinal Surgery, Tokyo Medical and Dental University, 7. Department of Gastrointestinal Medical Oncology, National Cancer Center Hospital, 8. Department of Gastroenterological Surgery, Nagoya City University Graduate School of Medical Science, 9. Department of Gastroenterological Surgery, Osaka University Graduate School of Medicine, 10. Department of Oncology and General Medicine, The Institute of Medical Science Hospital, The University of Tokyo, 11. Department of Gastric Surgery, National Cancer Center Hospital, 12. Division of Gastric Surgery, Shizuoka Cancer Center

**Corresponding author:** Takeshi Kawakami

Division of Gastrointestinal Oncology, Shizuoka Cancer Center, Sunto-gun, Nagizumi-cho, Shimonagakubo 1007, Shizuoka 411-0934, Japan

**E-mail:** t.kawakami@scchr.jp

Supplementary Table S1. Comparison between recommended and actual doses

|  | Actual dose (mg/day) | | |
| --- | --- | --- | --- |
| Recommended dose by  the formula (mg/day) | 80  (N = 55) | 100  (N = 257) | 120  (N = 374) |
| 80 | 53 | 159 | 14 |
| 100 | 2 | 85 | 151 |
| 120 | 0 | 10 | 166 |
| 160 | 0 | 2 | 38 |
| 200 | 0 | 1 | 4 |
| 240 | 0 | 0 | 1 |

　　　underdose, equal dose, overdose
